# Using Genetically Encoded Voltage Indicators (GEVIs) to Study the Input-Output Transformation of the Mammalian Olfactory Bulb

**DOI:** 10.3389/fncel.2019.00342

**Published:** 2019-07-31

**Authors:** Douglas A. Storace, Lawrence B. Cohen, Yunsook Choi

**Affiliations:** ^1^Department of Biological Science, Florida State University, Tallahassee, FL, United States; ^2^Department of Cellular and Molecular Physiology, Yale School of Medicine, Yale University, New Haven, CT, United States; ^3^Center for Functional Connectomics, Korea Institute of Science and Technology, Seoul, South Korea

**Keywords:** ArcLight, odors, olfactory bulb, mouse, perception, GEVIs, GECIs

## Abstract

Genetically encoded voltage indicators (GEVIs) are fluorescent protein reporters of membrane potential. These tools can, in principle, be used to monitor the neural activity of genetically distinct cell types in the brain. Although introduced in 1997, they have been a challenge to use to study intact neural circuits due to a combination of small signal-to-noise ratio, slow kinetics, and poor membrane expression. New strategies have yielded novel GEVIs such as ArcLight, which have improved properties. Here, we compare the *in vivo* properties of ArcLight with Genetically Encoded Calcium Indicators (GECIs) in the mouse olfactory bulb. We show how voltage imaging can be combined with organic calcium sensitive dyes to measure the input-output transformation of the olfactory bulb. Finally, we demonstrate that ArcLight can be targeted to olfactory bulb interneurons. The olfactory bulb contributes substantially to the perception of the concentration invariance of odor recognition.

## Introduction

Traditional optical imaging techniques using intrinsic signals (Blasdel and Salama, 1986; Grinvald et al., 1986) or organic dyes for measuring voltage (Davila et al., 1973) and calcium (Brown et al., 1975; Tsien, 1980), have limited ability to distinguish the cell types contributing to the signal, except in a few special cases (Tsau et al., 1996; Wenner et al., 1996; Friedrich and Korsching, 1997; Wachowiak and Cohen, 2001). In contrast, protein sensors of neural activity can be genetically targeted to different cell types. Genetically encoded voltage indicators (GEVI) optically report membrane potential in targeted cell types.

GEVIs have been improved in recent years (e.g., Jin et al., 2012; St-Pierre et al., 2014; Gong et al., 2015) and as a result they are starting to be used to answer neurobiological questions. ArcLight is a GEVI that can detect action potentials in cultured mammalian neurons (Jin et al., 2012), *in vivo* in *Caenorhabditis elegans* (Wooltorton et al., 2013) and *Drosophila* (Cao et al., 2013), and population voltage signals in mice (Storace et al., 2015, 2016; Borden et al., 2017; Storace and Cohen, 2017). Bando et al. (2019) compared ArcLight to other GEVIs and reported that ArcLight had the largest signal-to-noise ratio in both 1 and 2-photon imaging from the *in vivo* mouse brain.

In the first section of the paper we show that both the GEVIs and the voltage processes that they follow are faster than the GECIs and the calcium concentration changes that they follow. In the second section of the paper we describe the use of GEVIs and organic calcium dyes to determine the input-output transformation of the mammalian olfactory bulb. Comparing the input and output of a brain region defines the function(s) carried out by that region. In the third section of the paper we demonstrate that ArcLight can be targeted to an olfactory bulb interneuron. Measurements from different bulb cell types may help determine the mechanisms that shape the input-output transformations.

Typically an odorant is perceived as the same over a substantial range of odorant concentrations (Gross-Isseroff and Lancet, 1988; Krone et al., 2001; Uchida and Mainen, 2007; Homma et al., 2009). This perceptual concentration invariance is essential for animals, including mammals, that use odor to find food, or mates, or family members, or avoid predators, or to make the correct food aversion associations. The input from the nose to the olfactory bulb is a confound of odor quality and odor concentration (Rubin and Katz, 1999; Wachowiak and Cohen, 2001; Bozza et al., 2004). It was not known where in the olfactory sensory system this confound is disambiguated. Each individual olfactory bulb output mitral cell has a very large axon that reaches 12 different olfactory brain regions (Igarashi et al., 2012). Presumably it is in the olfactory bulb and/or in one or more of these cortical areas that this confound is unraveled.

In principle, the input-output comparison can be made using many single cell electrical or optical recordings but this process typically provides only a limited sampling from each animal. An alternative, used in the experiments described below, is to make population recordings with glomerular instead of cellular resolution. In the mouse each glomerulus receives the axon terminals from ∼1,000 olfactory receptor cells in the nose and ∼25 dendritic tufts from output mitral/tufted cells. Simultaneous *in vivo* sampling the odorant responses from 20 glomeruli thus includes the activity of ∼20,000 input cells and ∼500 output cells thereby reducing the sampling noise that occurs when recording from only a few neurons in each preparation. Using glomerular resolution gives up individual cell information in favor of the average response of the populations of the input and output neurons. Measuring population signals typically involves lower temporal resolution recordings and together with the averaging of responses from many neurons results in larger signal-to-noise ratios.

Optical measurements can simultaneously record the input and output in the same glomerulus by using indicators with different excitation or emission spectra, one in the input neurons and the other in the output cells.

Individual neuron recordings with GECIs are well established while single neuron recordings with GEVIs are just beginning. These are described elsewhere (Gong et al., 2015; Lou et al., 2016).

This paper emphasizes population GEVI measurements from the *in vivo* mouse olfactory bulb using ArcLight. Optical recordings from other preparations (Cao et al., 2013; Wooltorton et al., 2013) or using other GEVIs are described elsewhere (Knopfel et al., 2015; St-Pierre et al., 2015; Storace et al., 2015; Kim et al., 2016; Nakajima et al., 2016; Yang et al., 2016; Xu et al., 2017; Deo and Lavis, 2018; dam et al., 2019).

## Materials and Methods

### Surgical and Imaging Procedures

All experiments were performed in accordance with relevant guidelines and regulations, including a protocol approved by the Institutional Animal Care and Use Committees of Yale University. For all surgical procedures, male or female adult (40–100 days old) mice were anesthetized with a mixture of ketamine (90 mg kg^–1^) and xylazine (10 mg kg^–1^). Anesthesia was supplemented as needed to maintain areflexia, and anesthetic depth was monitored periodically via the pedal reflex. Animal body temperature was maintained at approximately 37.5°C using a heating pad placed underneath the animal. For recovery manipulations, animals were maintained on the heating pad until awakening. Local anesthetic (1% bupivacaine, McKesson Medical) was applied to all incisions. Respiration was recorded with a piezoelectric sensor.

For virus injections, a small (<1 mm) craniotomy was performed and AAV1 expressing ArcLight or GCaMP (injection volumes between 0.2 and 2 μl) was injected slowly over 10–15 min using a glass capillary (tip diameter 8–15 μm) ∼500 μm below the surface of the bulb. The ArcLight virus was generated at the Penn Vector Core and had a titer of 4.6e12 genome copies (GC) ml^–1^. The GCaMP3 and GCaMP6f virus were acquired from the Penn Vector Core and had titers of 4.7e13 GC ml^–1^ and 4.3e13 GC ml^–1^, respectively. We waited at least 10 days for expression of the virus prior to performing optical measurements.

The venerable, but not very reliable, method first described by Wachowiak and Cohen (2001) was used to load calcium dye into the olfactory sensory neurons. Mice were anesthetized, placed on their back, and an 8 μl mixture of 8/0.2% calcium dye/Triton-X was drawn into a flexible plastic syringe, which was inserted ∼10 mm into the nasal cavity. Using a Hamilton syringe, four injections of 2 μl of the dye/triton mixture was infused into the nose every 3 min. Mice were allowed to recover for at least 4 days prior to optical measurements. The organic calcium dye Fura dextran (F-3029) was from Thermo Fisher Scientific, Waltham, MA, United States. Cal-590 dextran is from AAT Bioquest (Sunnyvale, CA). The excitation spectra of these calcium dyes are well separated from that of many GEVIs and GECIs.

For epifluorescence or 2-photon imaging fluorescence measurements, mice were anesthetized, and the bone above both of the hemi-bulbs was either thinned or removed. The exposure was covered with agarose and then sealed with a glass coverslip.

Epifluorescence imaging was performed by illuminating the dorsal surface of the bulb with 150 W Xenon arc lamp (Opti Quip) on a custom antique Leitz Ortholux II microscope. The light was reflected by a 515 nm long-pass dichroic mirror before being delivered to the sample via an objective lens (various, typically a 4× 0.16NA or 10× 0.4NA lens). The fluorescence emission above 530 nm was recorded with a NeuroCCD-SM256 camera (RedShirtImaging, Decatur, GA, United States) with 2 × 2 binning at 40 or 125 Hz.

Two-photon imaging was performed with a modified MOM two-photon laser-scanning microscope (Sutter Instruments). 2-photon excitation was achieved using a Coherent Discovery laser (wavelengths between 940 and 980 nm), and laser scanning was performed with an 8 kHz resonant scanner (Cambridge Technology). Fluorescence was passed through a 510/84 nm bandpass filter and detected with a GaAsP PMT (Hamamatsu, Japan). Power delivered to the sample ranged from 75 to 140 mW as determined using a power meter (Thorlabs) placed underneath the microscope objective.

### AAV Vector

**FIGURE 1:**
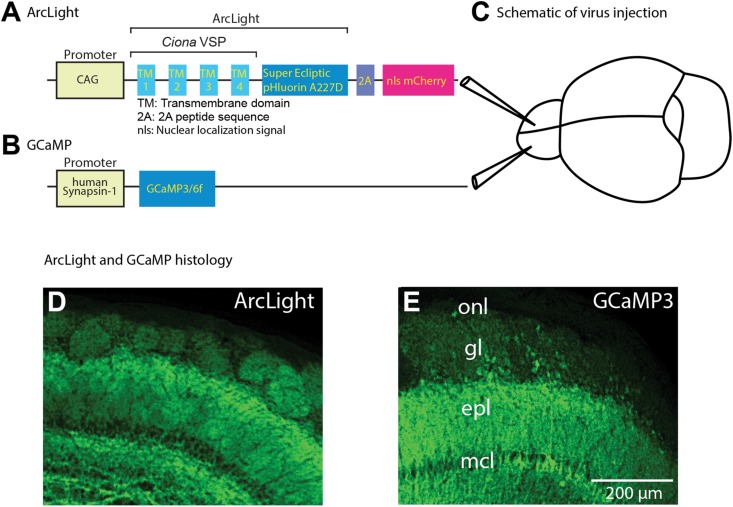
Schematic of viral vector constructs, virus injections to target both olfactory bulbs, and histological examination of ArcLight and GCaMP fluorescence. **(A,B)** Schematic drawing of the panel **(A)** ArcLight and **(B)** GCaMP3/6f AAV1 constructs. **(C)** ArcLight and GCaMP AAV vectors were injected into opposite hemibulbs of the same mouse between 10 and 50 days prior to imaging. **(D,E)** Confocal histological images demonstrate the expression patterns resulting from injecting the ArcLight **(D)** and GCaMP3 AAV vectors **(E)**. The scale bar in k also applies to h. onl, olfactory nerve layer; gl, glomerular layer; epl, external plexiform layer; mcl, mitral cell layer. Modified from Storace et al. (2015).

ArcLight A242-2A-nls-mCherry was constructed by fusing ArcLight A242 (Jin et al., 2012) with the self cleaving 2A peptide sequence followed by nuclear localized mCherry (Kim et al., 2012). To allow constitutive expression of the voltage sensor under the CAG promoter in wild type (C57BL/6) mice, ArcLight A242-2A-nls-mCherry was inserted into the aavCAG Jx vector (Gene bank JN898959) in a forward orientation. An adeno-associated virus serotype 1 (AAV1) of the ArcLight construct was produced at the Penn Vector Core at the University of Pennsylvania. For experiments with GCaMP3 and GCaMP6f, we used AAV1s expressing the gene under the human synapsin 1 (Figure 1B) promoter (#AV-1-PV1627 and AV-1-PV2822 purchased from the Penn Vector Core). For the TH-Cre targeting experiments, a Cre-dependent ArcLight virus was used (AV1.EF1a.ArcLight.DIO, Penn Vector Core).

### Transgenic Animals

Protocadherin 21 (Pcdh21) (Nagai et al., 2005) embryos were acquired from RIKEN BioResource Center (No. RBRC02189) and recovered by the Yale Genome Editing Center. OMP-GCaMP3 transgenic mice were a gift from T. Bozza. TH-Cre transgenic line were a gift from Matthew McGinley (Jax Stock #008601). Thy1-GCaMP6f GP5.11 transgenic mice were acquired from Jax (Stock #024339). Tbx21-Cre transgenic mice were acquired from Jax (Stock #024507) and crossed to a floxed GCaMP6f reporter transgenic mouse (Jax Stock #024105). Animals used in the study were confirmed to express the sensor via genotyping by Transnetyx (Cordova, TN, United States), and by visual inspection (*in vivo* and histologically).

### Data Analysis

For all experiments, odorant-evoked signals were collected in consecutive (1–20) trials separated by a minimum of 45 s. Individual trials were manually inspected and were discarded if they exhibited obvious movement artifact. Trials were averaged after being aligned to the time of the first inspiration following the odorant presentation. Individual glomeruli were visually identified via a frame subtraction analysis that identified stimulus activated glomeruli. The activation maps were generated by subtracting the temporal average of the 1–2 s preceding the stimulus from a 1 s temporal average at the signal peak using frame subtraction in NeuroPlex (RedShirtImaging). Spatial filtering is indicated in the figure legend. The response maps were depixelated for display. Response amplitudes were calculated as the difference between the peak of the response, and the 1–2 s period preceding the stimulus. Fluorescence signals were converted to ΔF/F by dividing the spatial average of each glomerulus by resting fluorescence (the average of the time period prior to the odor presentation).

The input and output measurements in Figure 5 were performed alternatively using Fura dextran and ArcLight. The 0.04% odor concentration did not evoke any detectable input glomerular response, and was subtracted from the input for the traces and values in (C) and (D). The evoked signal size for each concentration was normalized to the signal evoked at 11% of saturated vapor. To illustrate the similarity of the output maps, in Figure 5 each output response map was scaled so that all five maps had the same maximum and minimum intensity values. The input map for 11% saturated odor was scaled to have the same maximum and minimum brightness values as the five output maps. The input maps for the lower odorant concentrations were first scaled by the same scaling factors that were used for the output maps and are shown using the same maximum and minimum brightness scale as the 11% odorant map.

The signal-to-noise analysis was performed by measuring the amplitude of the ArcLight response to the first sniff of the odorant vs. the response immediately preceding the odorant. This analysis was performed in 10 preparations in averaged trials that were aligned to the first sniff following the odorant. An ROI that evoked the largest signal for that trial was selected for each preparation.

For the 2-photon input and output measurements in Figure 7, glomerular responses were normalized to the signal evoked at the highest odorant concentration (6% of saturated vapor). The responses from all identified individual glomeruli were measured (as in Figure 7B), and averaged together to form the values plotted in Figure 7C. Statistical significance for Figure 7 was measured using a Wilcoxon rank sum test comparing the average of input vs. output preparations at each concentration.

For the ArcLight measurements from TH expressing interneurons in Figure 8, functional signals were measured in 5 preparations, of which histology was processed and examined to confirm expression in the expected locations in 4 of them. The activity maps in Figure 8B are scaled to the minimum and maximum values evoked by the highest odorant concentration. The data in Figure 8D come from concentration series performed in 3 of the preparations. For a given odor, all of the activated glomeruli were identified, measured and then averaged together for each odor concentration to give each data point. The ΔF/F values were normalized to the highest odor concentration used for that preparation (11% of saturated vapor). Responses to 3 odorants were measured in one preparation, only one odorant was used in the other two preparations. The input and output data in Figure 8D (red and black lines) are taken from Storace and Cohen (2017). The statistical comparison of the data in Figure 8D was performed using a Wilcoxon rank sum test, and similar results were obtained whether the comparisons were performed across preparations (*N* = 3 independent TH measurements), across odor presentations (5 independent TH measurements), or when collapsing all normalized values to the lower odor concentrations (yielding 9 independent TH measurements).

### Odorant Stimuli and Delivery

Odorants (Sigma-Aldrich) were diluted from saturated vapor with cleaned air using a flow dilution olfactometer (Vucinic et al., 2006). The olfactometer provided a constant flow of air blown over the nares. Through a separate set of tubing, odorants were constantly injected into the olfactometer, but sucked away via a vacuum that was switched off during odorant presentation. Different odorants were delivered using separate Teflon tubing. Odorants were delivered at different concentrations (between 0.1 and 6% of saturated vapor). Ethyl tiglate, methyl valerate, 2-heptanone, a mixture of the 3 odorants, and n-butyl acetate were used in these experiments. The time course and relative concentrations of odorants presented by the olfactometer was confirmed using a photo-ionization detector (Aurora Scientific Inc., Aurora, ON, Canada) at the beginning of experiments.

## Results and Discussion

### Comparing *in vivo* GEVI and GECI Signals From AAV Transfected Mouse Mitral/Tufted Cells

ArcLight was used to record the population voltage signals from mitral and tufted cell dendritic tufts in each glomerulus. ArcLight was expressed using an adeno-associated virus serotype 1 (AAV1) that co-expressed nuclear localized mCherry to facilitate identification of the transduced neurons (Figure 1A). GECIs GCaMP3 and GCaMP6f were similarly expressed via AAV transduction. Sensor expression was examined by comparing the fluorescence of the GEVIs and GECIs histologically. The ArcLight (Figure 1D) and GCaMP3 (Figure 1E) vectors had similar expression patterns in the external bulb layers, and were both largely selective for mitral and tufted neurons (Figures 1D,E). We presume that the labeled glomeruli are the result of sensor expression in the mitral/tufted neuron primary dendritic tufts. These AAV1 vectors were selective for restricted cell populations in the bulb: this specificity may be due to a combination of the AAV serotype and the promoter selectivity.

Different odorants evoke distinct spatial and temporal patterns of glomerular activation in the mouse olfactory bulb (Rubin and Katz, 1999; Wachowiak and Cohen, 2001; Spors and Grinvald, 2002). AAV vectors carrying the genes for ArcLight and GCaMP3 or GCaMP6f (Figures 1A,B) were injected into separate bulb hemispheres (Figure 1C) between 10 and 50 days prior to optical measurements. Expression tended to be widespread in the injected hemispheres.

The ArcLight voltage signals were compared with those from the GECIs GCaMP3 (Tian et al., 2009) and GCaMP6f (Chen et al., 2013) using simultaneous recordings from opposite olfactory bulb hemispheres. Odor-evoked responses could be detected in the glomerular layer in single trials from all three sensors. ArcLight’s temporal kinetics were substantially faster than both GCaMPs but the signal-to-noise ratios were lower.

**FIGURE 2:**
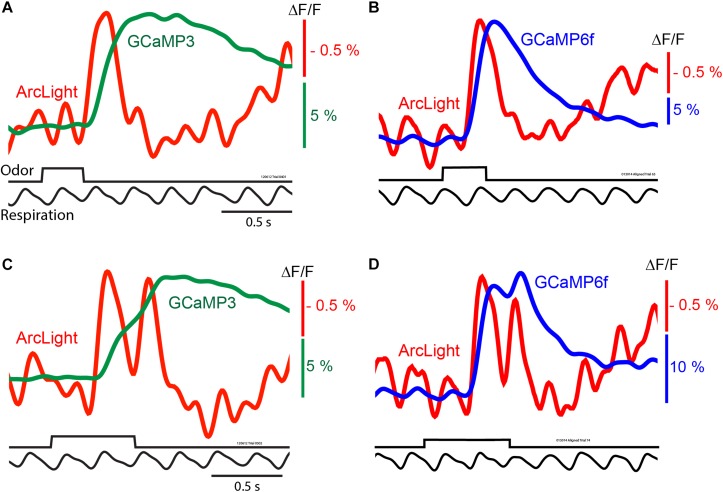
*In vivo* measurements of odorant responses in the mouse olfactory bulb using the GEVI ArcLight and two GCaMPs. Left panels **(A,C)** comparing ArcLight and GCaMP3 signals from opposite olfactory bulbs in the same preparation in response to odorant presentations lasting one and two breaths. Right panels **(B,D)** comparing ArcLight and GCaMP6f in response to odorant presentations lasting one and two breaths. The ArcLight signals are substantially faster than those of the GCaMPs. Modified from Storace et al. (2015).

Odorants presented for 0.3 s often coincided with a single inhalation, which resulted in ArcLight and GCaMP3 (Figure 2A, red vs. green trace) or ArcLight and GCaMP6f (Figure 2B, red vs. blue trace) fluorescence signals that were easily detected in single trials. Depolarizations measured with ArcLight and increases in calcium measured with the GCaMPs are shown as upward signals.

The ArcLight and GCaMP signals had different amplitudes, time courses and signal-to-noise ratios. While ArcLight had a much smaller fractional fluorescence change (ΔF/F) than either GCaMP, ArcLight had a faster onset, rise time, and decay (Storace et al., 2015). With a longer odorant pulse that elicits two odorant inhalations (Figures 2C,D) the ArcLight signal returns almost all the way to the baseline between the two inhalations while the GCaMP signals either have only an inflection (Figure 2C) or a modest return toward the baseline (Figure 2D).

While the signal-to-noise ratios of the ArcLight signals are relatively large, the ArcLight signals in Figure 2 appear noisier than those of the GCaMPs. We attempted to quantify the ArcLight signal-to-noise ratio by measuring the difference between the odor-evoked change, and the pre-odor oscillations. On average the largest signal evoked by an odor was 1.83% (±0.17), and the baseline oscillation was 0.47% (±0.05). The fold-change of the odor vs. baseline was 4.07 (±0.28). However some of this baseline “noise” is actually a real respiratory signal which is emphasized more in the ArcLight signals because of the faster ArcLight response (Figures 2B,D). We compared the timing of baseline ArcLight and GCaMP signals. Consistent with the notion that the baseline signals are physiological and not movement artifacts, the ArcLight signals preceded the GCaMP signals.

The results from repeated imaging trials were relatively consistent (Storace et al., 2015), which demonstrates that substantial photo-bleaching or photodynamic effects were not present with either ArcLight or the GCaMPs at the incident light intensities which we used. Our comparison of intrinsic signal activity from injected and control hemispheres did not detect a pharmacological effect of ArcLight on normal bulb function. However, continued attention to the possibility of photodynamic and phototoxic effect is desirable. The ArcLight signals were sufficiently large and fast, which made them clearly distinct from the intrinsic light scattering and flavoprotein autofluorescence signals.

### Comparison of ArcLight and GCaMP Sensor Response Speed

The differences in time course and respiration coupling that we found between ArcLight and the two GCaMPs are likely due to a combination of differences in the time courses of the cellular voltage and calcium changes as well as the response speeds of the different sensors. To further understand the differences in signal time courses, we compared the temporal kinetics of the three kinds of protein sensors.

**FIGURE 3:**
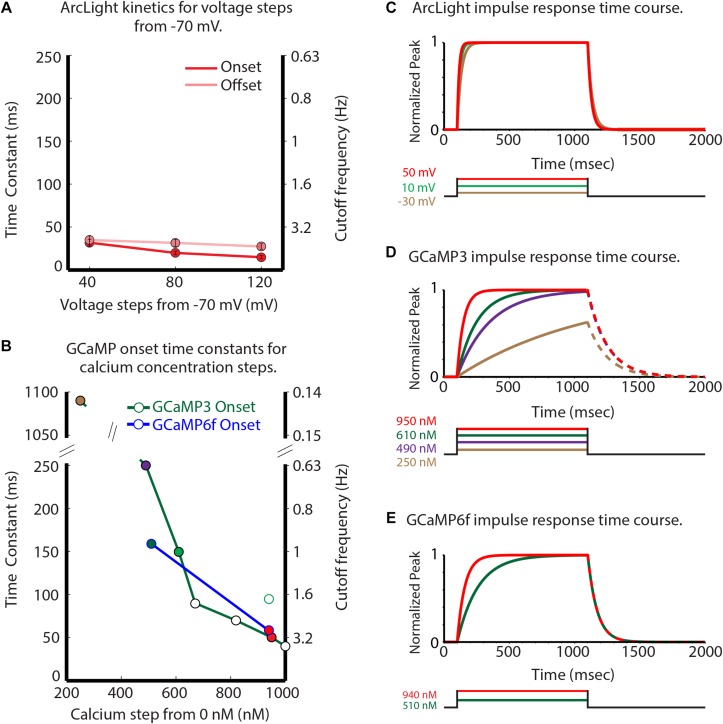
Comparison of the GEVI and GECI sensor kinetic properties. Experimentally measured ArcLight values for different depolarizing steps are compared with published and personally communicated values for the GECIs GCaMP3 and GCaMP6f. GCaMP3 and GCaMP6f are considerably slower in their onset response to calcium changes **(B)** than ArcLight is to membrane potential changes. **(A)** ArcLight onset (red) and offset (pink) time constants in response to different depolarizing steps from –70 mV in HEK293 cells. **(B)** The GCaMP3 (green line), and GCaMP6f (blue line) onset time constants in response to different calcium steps (Kim et al., personal communication). **(C–E)** Normalized impulse responses for ArcLight **(C)**, GCaMP3 **(D)**, and GCaMP6f **(E)** to physiologically relevant steps of voltage and calcium. The GCaMP offset rates in panels **(D,E)** are shown as dashed lines to indicate the possibility that they could be sensitive to the magnitude of the calcium change. Modified from Storace et al. (2015).

ArcLight had onset and offset time constants of 20 and 30 m s, respectively, for 80 mV depolarizing steps in HEK293 cells (Figure 3A). GCaMP3 has onset time constants of 1100 and 250 m s for calcium steps to 250 and 490 nM (Figure 3B, green line, time constants of 1100 and 250 m s) (Akerboom et al., 2012). GCaMP6f is about 50% faster for calcium concentration steps to ∼ 510 and ∼ 940 nM (Figure 3B, blue line). Figures 3C–E compares the temporal responses for ArcLight (C), GCaMP3 (D), and GCaMP6f (E) for selected steps of voltage and calcium. The GCaMP3 and GCaMP6f decay constants are reported to be ∼150 m s (Sun et al., 2013) and 71 m s (Kim et al., personal communication), respectively. These observations were done when starting at a high calcium concentration, however the effect of different calcium steps on these GECI decay times has not been reported. Clearly, ArcLight has faster kinetics than either of the two GCaMPs, which may explain some of its improved ability to detect individual inspiratory responses.

The ArcLight signal likely reflects the average of all of the mitral/tufted dendritic processes in the glomerular region of interest. Electrode measurements have shown that individual mitral and tufted cells can produce action potentials that are tightly linked to the respiration cycle (Dhawale et al., 2010; Carey and Wachowiak, 2011; Shusterman et al., 2011). Thus, our ArcLight measurements appear to accurately reflect the respiration driven envelope of mitral/tufted cell spiking activity (Rojas-Libano et al., 2014).

The onset and decay kinetics of the GCaMP sensor responses to step changes in calcium result from the calcium binding and unbinding rates. The mechanism generating the ArcLight signal results from a dimerization of the super ecliptic pHluorin of two ArcLight molecules which is then affected by the movement of S4 in the voltage sensitive domain (Kang and Baker, 2016). A pH fluorescent protein like super ecliptic pHluorin is necessary but the ArcLight signal is not the result of a change in pH (Han et al., 2014; Kang and Baker, 2016).

### Determining the Olfactory Bulb Input-Output Transformation

**FIGURE 4:**
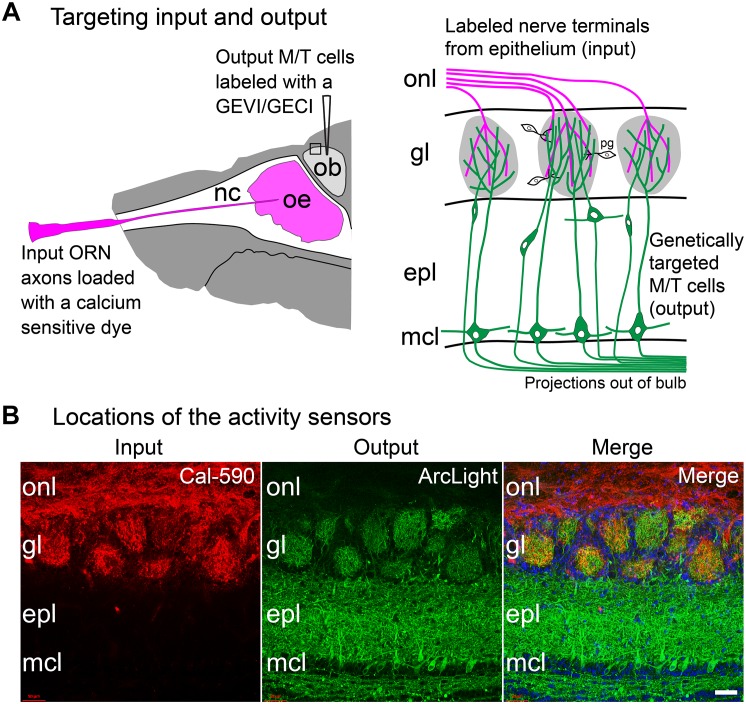
Approach to measure both the input and output of the same glomeruli in the mouse olfactory bulb. **(A)** Experimental approach: Left, the olfactory receptor neuron nerve terminal input is labeled via nasal infusion of an organic calcium sensitive dextran dye together with a low concentration of Triton-X. In the same preparation, GEVIs or GECIs were targeted to mitral and tufted output cells using cre-dependent AAVs in a transgenic mouse that expressed cre recombinase in those cells. Right, input and output can be measured independently from the same glomerulus by using activity sensors with substantially different excitation or emission spectra. The signals from the two cell types can be distinguished by changing the excitation or emission wavelengths. **(B)** A doubled labeled histological section showing targeting to input vs. output. In this section, Cal-590 dextran was anatomically targeted to the olfactory receptor nerve terminal input (left) and ArcLight was genetically targeted via AAV injection to the mitral and tufted cell output (middle). The merged image is shown on the right. Both sensors are present in each glomerulus. onl, olfactory nerve layer; gl, glomerular layer; epl, external plexiform layer; mcl, mitral cell layer. Scale bar in panel **(B)**, 50 μm. Modified from Storace and Cohen (2017).

An approach was developed to measure both the input and output of the same glomeruli in the mouse olfactory bulb. Spectrally distinct sensors of neural activity were used; one in the olfactory receptor neurons (input), the other in the mitral and tufted cells (output). This approach employed both anatomical and genetic targeting. Olfactory receptor neurons (input) were labeled via nasal infusion with an organic calcium sensitive dye (Figure 4A; Friedrich and Korsching, 1997; Wachowiak and Cohen, 2001). In the same preparation, GEVIs or GECIs were targeted to mitral and tufted (output) cells using cre-dependent AAVs in a transgenic mouse (Protocadherin 21) that expresses cre recombinase in the mitral and tufted cells (Figure 4A; Nagai et al., 2005). Appropriate targeting of the sensors in the expected locations was confirmed via histological examination (Figure 4B).

Because the input and output sensors had substantially different excitation spectra, input vs. output signals could be measured in the same glomeruli by changing the excitation wavelength. Odor-evoked activity was measured using epi-fluorescence imaging across ∼2 log units of odorant concentration in freely breathing anesthetized mice. Input and output measurements were made from the same hemi-bulb using the calcium dye Fura dextran as the input sensor, and the protein voltage sensor ArcLight as the output sensor. The Fura dextran input signal was imaged using 380 nm excitation light. The ArcLight output signals were measured with an excitation light of 480 nm. Fluorescence time courses were recorded from regions of interest corresponding to activated glomeruli. These time courses reflect the population average of the olfactory receptor neuron axon input activity or the population average of the mitral and tufted neuron output activity from individual glomeruli.

**FIGURE 5:**
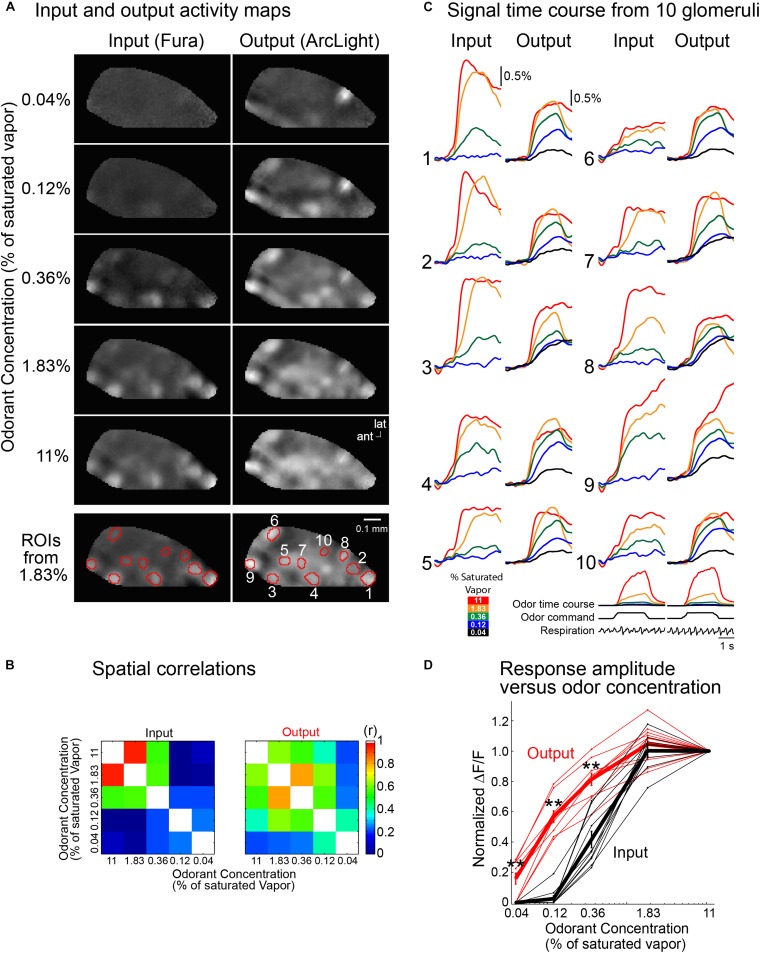
Comparing the input and output from ten glomeruli in the same bulb using Fura dextran (input) and the GEVI ArcLight (output). **(A)** Frame subtraction activity maps at five methyl valerate concentrations for input (left) and output (right). The output maps are much more similar to each other in comparison with the input maps. To illustrate the similarity of the output maps, each output response map was scaled so that all five maps had the same maximum and minimum brightness values. The input map for 11% saturated odor was scaled to have the same maximum and minimum brightness values as the five output maps. The input maps for the lower odorant concentrations were first scaled by the same scaling factors that were used for the output maps and are shown using the same maximum and minimum brightness scale as the 11% odorant map. The input map’s signals declined much more rapidly with decreasing odorant concentration. The selected glomeruli used for the time course results in panel **(C)** are indicated in the bottom panel (ROIs from 1.83%). **(B)** Spatial correlation of the 1.83% frame subtraction map with the four other odor concentrations. **(C)** Input and output traces of fluorescence vs. time for the ten glomeruli. The input signals decrease more dramatically than those of the output. The traces are low-pass filtered at 1 Hz. Odor time course (measured simultaneously with a photo-ionization detector), odor command pulse and respiration are shown under glomerulus 10. **(D)** Normalized peak fluorescence change vs. odorant concentration for the glomeruli in panel **(C)** (black, input; red, output). The activity maps in panel **(A)** and the traces in panel **(C)** are the average of 4–20 individual trials aligned to the first sniff following odor onset; ^∗∗^*p* < 0.001. ant, anterior; lat, lateral. Modified from Storace and Cohen (2017).

In the experiment illustrated in Figure 5, signal measurements were made from 10 glomeruli at five odorant concentrations. The output frame subtraction maps at the five odorant concentrations are more similar to each other than are the input maps (Figure 5A). The similarity of the input vs. output maps at different odor concentrations was quantified by performing a spatial correlation of each map with one another (Figure 5B). The output maps had a significantly higher spatial correlation than the input maps (*p* < 0.05, two-sample *t*-test). The signals from the 10 input and output glomeruli (Figure 5C, ROIs) also demonstrate that the input glomeruli have a considerably steeper function of odor concentration than the output glomeruli. The input exhibited no detectable signal at the lowest odorant concentration (0.04% saturated vapor, not shown), and very few had a signal at 0.12% saturated vapor. In contrast, an output signal was detected for all glomeruli at an odorant concentration of 0.04%.

The amplitudes of the odor-evoked input and output signals for each glomerulus were normalized to the response evoked by the highest odorant concentration (11% of saturated vapor). Responses from individual glomeruli are plotted as the thin lines in Figure 5D, and the mean evoked signals are shown in the thick lines (red for output; black for input). Reducing the concentration of the odor presentation from 11 to 0.12% reduced the input amplitude to decrease by more than 95%, while the output decreased by only 40% (*p* < 0.001). This result was similarly consistent in a population average across 13 preparations (Storace and Cohen, 2017). Thus both the output activity maps and the output signal size are much less concentration dependent than are the input maps and signals. The olfactory bulb contributes to the perception of concentration invariance of odor recognition.

**FIGURE 6:**
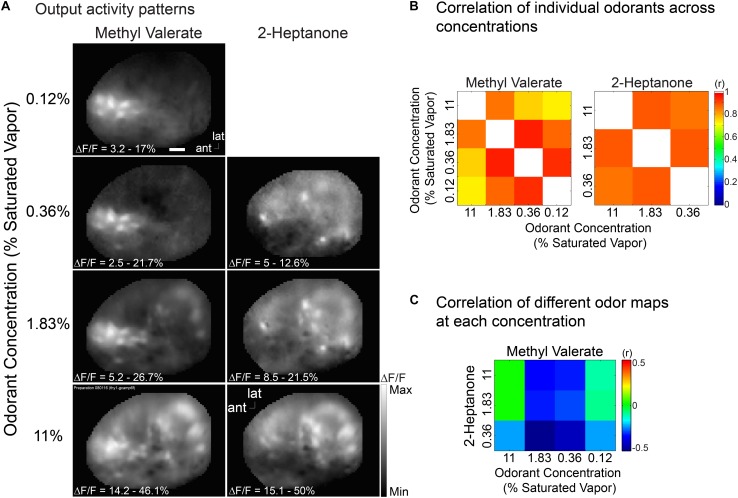
Output maps are relatively concentration invariant, but they are odorant specific. **(A)** Output maps in response to two odorants, methyl valerate and 2-heptanone, presented at several concentrations. **(B,C)** Output map correlations evoked by the same **(B)** and different **(C)** odorant. The output maps have a much higher correlation for the same odor than for different odors. This example is from a Thy1-GCaMP6f transgenic mouse in which GCaMP6f is selectively expressed in bulb output neurons (Dana et al., 2014). Similar results were obtained in two other preparations using Pcdh21-Cre transgenic mice. Scale bar in a 250 μm. ant, anterior; lat, lateral. Modified from Storace and Cohen (2017).

In another mouse we measured the concentration dependence of the output maps using two different odorants, methyl valerate and 2-heptanone. Figure 6A again shows that for each odorant the output maps do not change markedly over a substantial range of odorant concentration. Nonetheless the output maps are markedly different for the two odorants. Figures 6B,C show that the map correlations for the individual odorants are relatively large while the correlations across odorants are very small. Thus the bulb contributes to the perception of concentration invariance while maintaining an odorant specific output.

**FIGURE 7:**
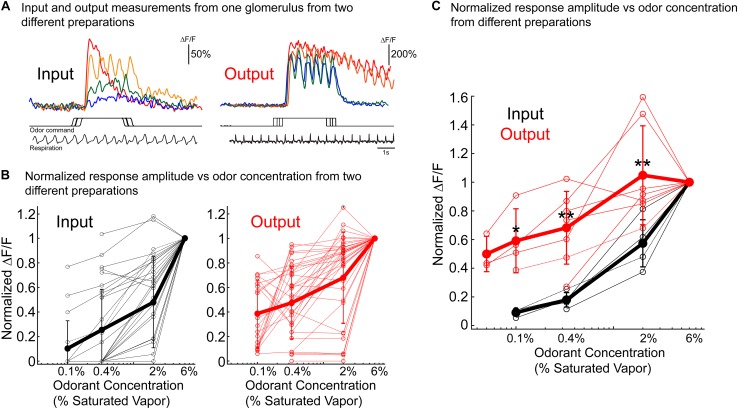
Concentration dependence of bulb input and output measured using 2-photon imaging. A different preparation was used for each measurement. OMP-GCaMP3 transgenic mice in which GCaMP3 is endogenously expressed in the olfactory receptor neurons was used for the input measurements. For the output measurements Thy1-GCaMP6f 5.11 or Tbx21-Cre mice that were crossed to a flox-GCaMP6f transgenic mouse. The output results were similar in the two transgenic lines. **(A)** Example results from single glomeruli from preparations in which glomerular input (left) and output (right) were measured in response to the same odor concentration range. The respiration trace is from the 2% single trial for both input and output. The traces in panel **(A)** are taken from single trials. The responses were similar across repeats taken 3 min apart. **(B)** Concentration vs. normalized ΔF/F for all glomeruli measured from the same two preparations used in panel **(A)** (Input: 31 glomeruli; Output: 37 glomeruli). Mean ± SEM is shown as the thick line. The output example in panels **(A,B)** is from a Thy1-GCaMP6f 5.11 preparation. **(C)** Population average concentration vs. normalized ΔF/F. All individual glomeruli were averaged for each preparation (thin lines). The results include measurements from five input preparations and seven output preparations. Wilcoxon rank sum test was used to compare the two distributions (ranksum in MATLAB) ^*^p < 0.05 and ^∗∗^*p* < 0.01. The quantification in panels **(B,C)** are from averaged trials.

One concern about the results illustrated in Figures 5C,D (and in similar figures in Storace and Cohen, 2017) is the possible contribution of the out-of-focus mitral/tufted lateral dendrite signals to the measurements. Accordingly, we have repeated the measurements of input and output concentration dependence using 2-photon microscopy where the depth-of-field is <10 microns instead of the >100 microns of wide-field microscopy. These experiments were carried out using different animals for each input and output measurement. Figure 7A illustrates the results from one glomerulus from two different animals. The input signals decline markedly as a function of concentration while the output signal is much less concentration dependent. Figure 7B shows a plot of signal size vs. odorant concentration for all of the identified glomeruli in the two preparations used in (a). The normalized response of the output was significantly higher than the input for all tested concentration (*p* < 0.05; Input: 31 glomeruli; Output: 37 glomeruli). Figure 7C shows that across a population the average input signal (*N* = 5 preparations; thick black line) declines much more rapidly than the average output signal (*N* = 7 preparations; thick red line) (Figure 7C) (*p* < 0.05 using a Wilcoxon rank sum test for all comparisons). The results in Figure 7 are very similar to those published earlier (Storace and Cohen, 2017) using wide-field microscopy and thus they alleviate the concern that the wide-field conclusion might have been in error because of contamination from mitral/tufted lateral dendrite signals.

We conclude that the olfactory bulb contributes to the perception that the quality of an odorant is considered the same over a range of concentrations. It has been hypothesized that the combination of odorant receptors that are activated by an odorant determines odor identity.

However, previous studies have found that the olfactory bulb glomerular input maps were a confound of odorant identity and concentration (Wachowiak and Cohen, 2001; Bozza et al., 2004). While the activated receptors are critical for odor identification, our results show that odorant identity is determined by the olfactory bulb output. The intensity information may be normalized in the olfactory bulb, and thus the bulb in part removes the qualitative effect of odorant concentration so that odor identity is represented in the output maps. However, the mitral and tufted cell population average still maintains some concentration dependence so that intensity information is not entirely discarded.

The signal-to-noise ratio in the Fura dextran input recordings has a threshold that is ∼5% of the largest signal. If the largest signal represents the activation of ∼1000 olfactory receptor neurons, then the smallest detectable signal would reflect the average activity of less than 50 receptor neurons. The activity of <50 receptor neurons is enough to generate substantial output signals and enough to enable odorant recognition (Homma et al., 2009). However, this argument assumes linearity of the sensor signal with calcium concentration, while calcium signals are typically nonlinear.

The possibility that the olfactory bulb could be involved in normalizing the well-described concentration dependence of the olfactory sensory neurons was proposed by Thomas Cleland (Cleland et al., 2007, 2011). Since that suggestion there is a growing body of supporting evidence across multiple species including *Drosophila* (Asahina et al., 2009), Zebrafish (Niessing and Friedrich, 2010), and mouse (Banerjee et al., 2015; Sirotin et al., 2015; Storace and Cohen, 2017; this paper). A strength of the results in Storace and Cohen (2017) is that the input and output of the olfactory bulb were measured in the same preparation (in some cases simultaneously) allowing a direct comparison to be made. Candidate mechanisms include different types of interneurons with distinct anatomical connections that could facilitate communication across glomeruli, and the ability of some cell types to bidirectionally modulate circuits (Yaksi and Wilson, 2010; Zhu et al., 2013; Banerjee et al., 2015). The results presented here and in other reports (Banerjee et al., 2015; Storace and Cohen, 2017; Bolding and Franks, 2018) also demonstrate that not all concentration information is discarded in the olfactory bulb. It seems likely that the olfactory bulb is performing computations that are critical for downstream targets (e.g., in the Piriform Cortex) to perform invariant object identification. Recent evidence has shown that while the olfactory bulb mitral/tufted cells are relatively concentration invariant, piriform cortex cells are even less sensitivity to concentration, a function that involves feedback from the olfactory cortex to the olfactory bulb (Bolding and Franks, 2018). Thus, concentration invariant odorant perception likely involves dynamic processing across multiple brain regions. Nonetheless, it is clear that the olfactory bulb is involved in a critical processing step to normalize the highly concentration dependent olfactory bulb input.

### *In vivo* ArcLight Signals From a Tyrosine Hydroxylase-Cre Transgenic Mouse

**FIGURE 8:**
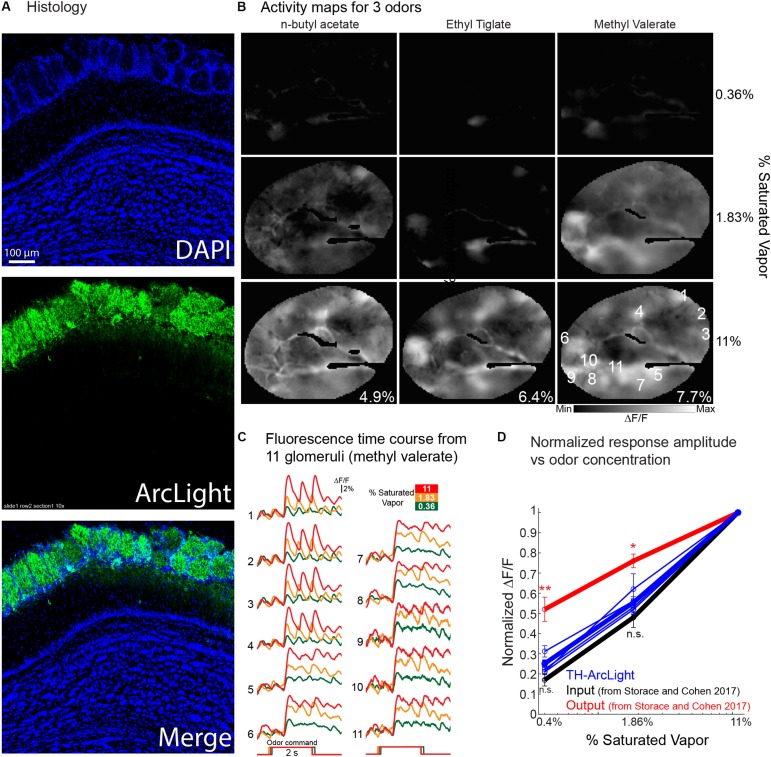
Odor-evoked *in vivo* recordings of the GEVI voltage indicator ArcLight from tyrosine hydroxylase (TH) expressing olfactory bulb juxtaglomerular interneurons. **(A)** Histology of cells expressing ArcLight. **(B)** Activity maps evoked by three different odorants at three concentrations. Maps are from an average of 2–4 individual trials that were aligned to the onset of the signal, and were generated by subtracting the ∼0.5–1 s during the odor stimulus from the 2 s prior to the stimulus onset. The images were sharpened using high-pass Gaussian filter with a kernel size of 61 × 61 pixels. Activity maps are scaled to the minimum and maximum values evoked by the highest odor concentration. The maximum ΔF/F value for each odor is included in the bottom right of the highest odor concentration map. **(C)** Fluorescence time courses from the 11 glomeruli identified in the middle concentration methyl valerate image in panel **(B)**. The trials are aligned to the onset of the response. **(D)** Normalized response amplitude vs. odor concentration for populations of glomeruli measured from 3 different preparations. TH-ArcLight measurements are from 5 different odorant trials across three preparations. Input and output measurements (red and black lines) are adapted from Storace and Cohen (2017). ^∗∗^*p* < 0.01 and ^*^p < 0.05.

Measuring the contribution of different olfactory bulb cell types may help reveal the mechanisms that shape the transformations occurring in the olfactory bulb. Here, we targeted ArcLight to bulb tyrosine hydroxylase (TH) interneurons with an AAV1 cre dependent ArcLight vector. The TH interneurons are restricted to the glomerular layer and include more than one interneuron type (Pignatelli et al., 2005; Pignatelli and Belluzzi, 2017). Figure 8A illustrates the DAPI and ArcLight expression. The ArcLight expression is restricted to cells and processes in the juxtaglomerular layer consistent with the possibility that the expression is restricted to TH periglomerular interneurons.

Activity maps for the odorant responses (Figure 8B) were made by subtracting imaging frames prior to the odorant stimulus from frames acquired during the response. Maps for three concentrations of three odorants are illustrated. These maps are very concentration dependent suggesting that they reflect the olfactory receptor cell input to the bulb rather that the mitral/tufted cell output (Banerjee et al., 2015). The time course of the ArcLight fluorescence changes at three concentrations from 11 glomeruli are shown in Figure 8C (single trials). Figure 8D is a plot of normalized response amplitude vs. odor concentration of the glomerular TH measurements, overlaid with the population input and output measurements from Storace and Cohen (2017).

The normalized TH concentration dependence was not statistically different than the input, and was significantly smaller than the output (*p* < 0.05; Wilcoxon rank sum). While these measurements are from a more limited dataset and performed in different animals, the result support other studies that have proposed that these cells carry the concentration dependent information from the bulb input. These signals may be transmitted throughout the bulb via long-range lateral connections that are found in many of these cells (Aungst et al., 2003; Zhu et al., 2013; Banerjee et al., 2015).

## Conclusion

In population measurements from mouse olfactory bulb glomeruli, the ArcLight signals provide important information about membrane potential changes and the perceptual computations carried out in the bulb. In the future we expect that the role of the olfactory bulb in other olfactory perceptions could be determined by using our approach to measure both the input and output. This strategy will help guide investigations into the function of the complex synaptic network of the olfactory bulb. Similar strategies as those used here could be used to understand the input-output transformation of other brain regions.

## Data Availability

The raw data supporting the conclusions of this manuscript will be made available by the authors, without undue reservation, to any qualified researcher.

## Ethics Statement

All experiments were performed in accordance with relevant guidelines and regulations, including a protocol approved by the Institutional Animal Care and Use Committees of Yale University.

## Author Contributions

All authors listed have made a substantial, direct and intellectual contribution to the work, and approved it for publication.

## Conflict of Interest Statement

The authors declare that the research was conducted in the absence of any commercial or financial relationships that could be construed as a potential conflict of interest.

## References

[B1] Akerboom J. Chen T. W. Wardill T. J. Tian L. Marvin J. S. Mutlu S. (2012). Optimization of a GCaMP calcium indicator for neural activity imaging. *J. Neurosci.* 32 13819–13840. 10.1523/JNEUROSCI.2601-12.2012 23035093 PMC3482105

[B2] Asahina K. Louis M. Piccinotti S. Vosshall L. B. (2009). A circuit supporting concentration-invariant odor perception in *Drosophila*. *J. Biol.* 8:9. 10.1186/jbiol108 19171076 PMC2656214

[B3] Aungst J. L. Heyward P. M. Puche A. C. Karnup S. V. Hayar A. Szabo G. (2003). Centre-surround inhibition among olfactory bulb glomeruli. *Nature* 426 623–629. 10.1038/nature02185 14668854

[B4] Bando Y. Sakamoto M. Kim S. Ayzenshtat I. Yuste R. (2019). Comparative evaluation of genetically encoded voltage indicators. *Cell Rep.* 26 802–813.e4. 10.1016/j.celrep.2018.12.088 30650368 PMC7075032

[B5] Banerjee A. Marbach F. Anselmi F. Koh M. S. Davis M. B. Garcia Da Silva P. (2015). An interglomerular circuit gates glomerular output and implements gain control in the mouse olfactory bulb. *Neuron* 87 193–207. 10.1016/j.neuron.2015.06.019 26139373 PMC4633092

[B6] Blasdel G. G. Salama G. (1986). Voltage-sensitive dyes reveal a modular organization in monkey striate cortex. *Nature* 321 579–585. 10.1038/321579a0 3713842

[B7] Bolding K. A. Franks K. M. (2018). Recurrent cortical circuits implement concentration-invariant odor coding. *Science* 361:eaat6904. 10.1126/science.aat6904 30213885 PMC6492549

[B8] Borden P. Y. Ortiz A. D. Waiblinger C. Sederberg A. J. Morrissette A. E. Forest C. R. (2017). Genetically expressed voltage sensor arcLight for imaging large scale cortical activity in the anesthetized and awake mouse. *Neurophotonics* 4:031212. 10.1117/1.NPh.4.3.031212 28491905 PMC5416966

[B9] Bozza T. Mcgann J. P. Mombaerts P. Wachowiak M. (2004). In vivo imaging of neuronal activity by targeted expression of a genetically encoded probe in the mouse. *Neuron* 42 9–21. 10.1016/s0896-6273(04)00144-8 15066261

[B10] Brown J. E. Cohen L. B. De Weer P. Pinto L. H. Ross W. N. Salzberg B. M. (1975). Rapid changes in intracellular free calcium concentration. *Biophys. J.* 15 1155–1160. 10.1016/S0006-3495(75)85891-7 1201331 PMC1334796

[B11] Cao G. Platisa J. Pieribone V. A. Raccuglia D. Kunst M. Nitabach M. N. (2013). Genetically targeted optical electrophysiology in intact neural circuits. *Cell* 154 904–913. 10.1016/j.cell.2013.07.027 23932121 PMC3874294

[B12] Carey R. M. Wachowiak M. (2011). Effect of sniffing on the temporal structure of mitral/tufted cell output from the olfactory bulb. *J. Neurosci.* 31 10615–10626. 10.1523/JNEUROSCI.1805-11.2011 21775605 PMC3159407

[B13] Chen T. W. Wardill T. J. Sun Y. Pulver S. R. Renninger S. L. Baohan A. (2013). Ultrasensitive fluorescent proteins for imaging neuronal activity. *Nature* 499 295–300. 10.1038/nature12354 23868258 PMC3777791

[B14] Cleland T. A. Chen S. Y. Hozer K. W. Ukatu H. N. Wong K. J. Zheng F. (2011). Sequential mechanisms underlying concentration invariance in biological olfaction. *Front. Neuroeng.* 4:21. 10.3389/fneng.2011.00021 22287949 PMC3251820

[B15] Cleland T. A. Johnson B. A. Leon M. Linster C. (2007). Relational representation in the olfactory system. *Proc. Natl. Acad. Sci. U.S.A.* 104 1953–1958. 10.1073/pnas.0608564104 17261800 PMC1794271

[B16] dam Y. Kim J. J. Lou S. Zhao Y. Xie M. E. Brinks D. (2019). Voltage imaging and optogenetics reveal behaviour-dependent changes in hippocampal dynamics. *Nature* 569 413–417. 10.1038/s41586-019-1166-7 31043747 PMC6613938

[B17] Dana H. Chen T. W. Hu A. Shields B. C. Guo C. Looger L. L. (2014). Thy1-GCaMP6 transgenic mice for neuronal population imaging in vivo. *PLoS One* 9:e108697. 10.1371/journal.pone.0108697 25250714 PMC4177405

[B18] Davila H. V. Salzberg B. M. Cohen L. B. Waggoner A. S. (1973). A large change in axon fluorescence that provides a promising method for measuring membrane potential. *Nat. New Biol.* 241 159–160. 10.1038/newbio241159a0 4512623

[B19] Deo C. Lavis L. D. (2018). Synthetic and genetically encoded fluorescent neural activity indicators. *Curr. Opin. Neurobiol.* 50 101–108. 10.1016/j.conb.2018.01.003 29454295

[B20] Dhawale A. K. Hagiwara A. Bhalla U. S. Murthy V. N. Albeanu D. F. (2010). Non-redundant odor coding by sister mitral cells revealed by light addressable glomeruli in the mouse. *Nat. Neurosci.* 13 1404–1412. 10.1038/nn.2673 20953197 PMC3208311

[B21] Friedrich R. W. Korsching S. I. (1997). Combinatorial and chemotopic odorant coding in the zebrafish olfactory bulb visualized by optical imaging. *Neuron* 18 737–752. 10.1016/s0896-6273(00)80314-1 9182799

[B22] Gong Y. Huang C. Li J. Z. Grewe B. F. Zhang Y. Eismann S. (2015). High-speed recording of neural spikes in awake mice and flies with a fluorescent voltage sensor. *Science* 350 1361–1366. 10.1126/science.aab0810 26586188 PMC4904846

[B23] Grinvald A. Lieke E. Frostig R. D. Gilbert C. D. Wiesel T. N. (1986). Functional architecture of cortex revealed by optical imaging of intrinsic signals. *Nature* 324 361–364. 10.1038/324361a0 3785405

[B24] Gross-Isseroff R. Lancet D. (1988). Concentration-dependent changes of perceived odor quality. *Chemical. Senses* 13 191–204. 10.1093/chemse/13.2.191

[B25] Han Z. Jin L. Chen F. Loturco J. J. Cohen L. B. Bondar A. (2014). Mechanistic studies of the genetically encoded fluorescent protein voltage probe arclight. *PLoS One* 9:e113873. 10.1371/journal.pone.0113873 25419571 PMC4242678

[B26] Homma R. Cohen L. B. Kosmidis E. K. Youngentob S. L. (2009). Perceptual stability during dramatic changes in olfactory bulb activation maps and dramatic declines in activation amplitudes. *Eur. J. Neurosci.* 29 1027–1034. 10.1111/j.1460-9568.2009.06644.x 19291227 PMC2762425

[B27] Igarashi K. M. Ieki N. An M. Yamaguchi Y. Nagayama S. Kobayakawa K. (2012). Parallel mitral and tufted cell pathways route distinct odor information to different targets in the olfactory cortex. *J. Neurosci.* 32 7970–7985. 10.1523/JNEUROSCI.0154-12.2012 22674272 PMC3636718

[B28] Jin L. Han Z. Platisa J. Wooltorton J. R. Cohen L. B. Pieribone V. A. (2012). Single action potentials and subthreshold electrical events imaged in neurons with a fluorescent protein voltage probe. *Neuron* 75 779–785. 10.1016/j.neuron.2012.06.040 22958819 PMC3439164

[B29] Kang B. E. Baker B. J. (2016). Pado, a fluorescent protein with proton channel activity can optically monitor membrane potential, intracellular pH, and map gap junctions. *Sci. Rep.* 6:23865. 10.1038/srep23865 27040905 PMC4878010

[B30] Kim J. Zhao T. Petralia R. S. Yu Y. Peng H. Myers E. (2012). mGRASP enables mapping mammalian synaptic connectivity with light microscopy. *Nat. Methods* 9 96–102. 10.1038/nmeth.1784 PMC3424517 22138823

[B31] Kim T. H. Zhang Y. Lecoq J. Jung J. C. Li J. Zeng H. (2016). Long-term optical access to an estimated one million neurons in the live mouse cortex. *Cell Rep.* 17 3385–3394. 10.1016/j.celrep.2016.12.004 28009304 PMC5459490

[B32] Knopfel T. Gallero-Salas Y. Song C. (2015). Genetically encoded voltage indicators for large scale cortical imaging come of age. *Curr. Opin. Chem. Biol.* 27 75–83. 10.1016/j.cbpa.2015.06.006 26115448

[B33] Krone D. Mannel M. Pauli E. Hummel T. (2001). Qualitative and quantitative olfactometric evaluation of different concentrations of ethanol peppermint oil solutions. *Phytother. Res.* 15 135–138. 10.1002/ptr.716 11268113

[B34] Lou S. Adam Y. Weinstein E. N. Williams E. Williams K. Parot V. (2016). Genetically targeted all-optical electrophysiology with a transgenic cre-dependent optopatch mouse. *J. Neurosci.* 36 11059–11073. 10.1523/jneurosci.1582-16.2016 27798186 PMC5098841

[B35] Nagai Y. Sano H. Yokoi M. (2005). Transgenic expression of cre recombinase in mitral/tufted cells of the olfactory bulb. *Genesis* 43 12–16. 10.1002/gene.20146 16106355

[B36] Nakajima R. Jung A. Yoon B. J. Baker B. J. (2016). Optogenetic monitoring of synaptic activity with genetically encoded voltage indicators. *Front. Synaptic. Neurosci.* 8:22. 10.3389/fnsyn.2016.00022 27547183 PMC4974255

[B37] Niessing J. Friedrich R. W. (2010). Olfactory pattern classification by discrete neuronal network states. *Nature* 465 47–52. 10.1038/nature08961 20393466

[B38] Pignatelli A. Belluzzi O. (2017). Dopaminergic neurones in the main olfactory bulb: an overview from an electrophysiological perspective. *Front. Neuroanat.* 11:7. 10.3389/fnana.2017.00007 28261065 PMC5306133

[B39] Pignatelli A. Kobayashi K. Okano H. Belluzzi O. (2005). Functional properties of dopaminergic neurones in the mouse olfactory bulb. *J. Physiol.* 564 501–514. 10.1113/jphysiol.2005.084632 15731185 PMC1464431

[B40] Rojas-Libano D. Frederick D. E. Egana J. I. Kay L. M. (2014). The olfactory bulb theta rhythm follows all frequencies of diaphragmatic respiration in the freely behaving rat. *Front. Behav. Neurosci.* 8:214. 10.3389/fnbeh.2014.00214 24966821 PMC4053074

[B41] Rubin B. D. Katz L. C. (1999). Optical imaging of odorant representations in the mammalian olfactory bulb. *Neuron* 23 499–511. 10.1016/s0896-6273(00)80803-x 10433262

[B42] Shusterman R. Smear M. C. Koulakov A. A. Rinberg D. (2011). Precise olfactory responses tile the sniff cycle. *Nat. Neurosci.* 14 1039–1044. 10.1038/nn.2877 21765422 PMC13348895

[B43] Sirotin Y. B. Shusterman R. Rinberg D. (2015). Neural coding of perceived odor intensity. *eNeuro* 2:ENEURO.0083-15 10.1523/ENEURO.0083-15.2015 PMC4672005 26665162

[B44] Spors H. Grinvald A. (2002). Spatio-temporal dynamics of odor representations in the mammalian olfactory bulb. *Neuron* 34 301–315. 10.1016/s0896-6273(02)00644-x 11970871

[B45] Storace D. Sepehri Rad M. Kang B. Cohen L. B. Hughes T. Baker B. J. (2016). Toward Better Genetically Encoded Sensors of Membrane Potential. *Trends Neurosci.* 39 277–289. 10.1016/j.tins.2016.02.005 27130905 PMC4852096

[B46] Storace D. A. Braubach O. R. Jin L. Cohen L. B. Sung U. (2015). Monitoring brain activity with protein voltage and calcium sensors. *Sci. Rep.* 5:10212. 10.1038/srep10212 25970202 PMC4429559

[B47] Storace D. A. Cohen L. B. (2017). Measuring the olfactory bulb input-output transformation reveals a contribution to the perception of odorant concentration invariance. *Nat. Commun.* 8:81. 10.1038/s41467-017-00036-2 28724907 PMC5517565

[B48] St-Pierre F. Chavarha M. Lin M. Z. (2015). Designs and sensing mechanisms of genetically encoded fluorescent voltage indicators. *Curr. Opin. Chem. Biol.* 27 31–38. 10.1016/j.cbpa.2015.05.003 26079047 PMC4553077

[B49] St-Pierre F. Marshall J. D. Yang Y. Gong Y. Schnitzer M. J. Lin M. Z. (2014). High-fidelity optical reporting of neuronal electrical activity with an ultrafast fluorescent voltage sensor. *Nat. Neurosci.* 17 884–889. 10.1038/nn.3709 24755780 PMC4494739

[B50] Sun X. R. Badura A. Pacheco D. A. Lynch L. A. Schneider E. R. Taylor M. P. (2013). Fast GCaMPs for improved tracking of neuronal activity. *Nat. Commun.* 4:2170. 10.1038/ncomms3170 23863808 PMC3824390

[B51] Tian L. Hires S. A. Mao T. Huber D. Chiappe M. E. Chalasani S. H. (2009). Imaging neural activity in worms, flies and mice with improved GCaMP calcium indicators. *Nat. Methods* 6 875–881. 10.1038/nmeth.1398 19898485 PMC2858873

[B52] Tsau Y. Wenner P. O’donovan M. J. Cohen L. B. Loew L. M. Wuskell J. P. (1996). Dye screening and signal-to-noise ratio for retrogradely transported voltage-sensitive dyes. *J. Neurosci. Methods* 70 121–129. 10.1016/s0165-0270(96)00109-4 9007751

[B53] Tsien R. Y. (1980). New calcium indicators and buffers with high selectivity against magnesium and protons: design, synthesis, and properties of prototype structures. *Biochemistry* 19 2396–2404. 10.1021/bi00552a018 6770893

[B54] Uchida N. Mainen Z. F. (2007). Odor concentration invariance by chemical ratio coding. *Front. Syst. Neurosci.* 1:3. 10.3389/neuro.06.003.2007 18958244 PMC2526272

[B55] Vucinic D. Cohen L. B. Kosmidis E. K. (2006). Interglomerular center-surround inhibition shapes odorant-evoked input to the mouse olfactory bulb in vivo. *J. Neurophysiol.* 95 1881–1887. 10.1152/jn.00918.2005 16319205

[B56] Wachowiak M. Cohen L. B. (2001). Representation of odorants by receptor neuron input to the mouse olfactory bulb. *Neuron* 32 723–735. 10.1016/s0896-6273(01)00506-2 11719211

[B57] Wenner P. Tsau Y. Cohen L. B. O’donovan M. J. Dan Y. (1996). Voltage-sensitive dye recording using retrogradely transported dye in the chicken spinal cord: staining and signal characteristics. *J. Neurosci. Methods* 70 111–120. 10.1016/s0165-0270(96)00108-2 9007750

[B58] Wooltorton J. R. He L. Salzberg B. M. Fang-Yen C. (2013). *In vivo* optical recording of action potentials in *C. elegans* muscles using arclight, a genetically expressed voltage sensitive fluorescent protein. *Biophys. J.* 104:340a 10.1016/j.bpj.2012.11.1889

[B59] Xu Y. Zou P. Cohen A. E. (2017). Voltage imaging with genetically encoded indicators. *Curr. Opin. Chem. Biol.* 39 1–10. 10.1016/j.cbpa.2017.04.005 28460291 PMC5581692

[B60] Yaksi E. Wilson R. I. (2010). Electrical coupling between olfactory glomeruli. *Neuron* 67 1034–1047. 10.1016/j.neuron.2010.08.041 20869599 PMC2954501

[B61] Yang H. H. St-Pierre F. Sun X. Ding X. Lin M. Z. Clandinin T. R. (2016). Subcellular imaging of voltage and calcium signals reveals neural processing in vivo. *Cell* 166 245–257. 10.1016/j.cell.2016.05.031 27264607 PMC5606228

[B62] Zhu P. Frank T. Friedrich R. W. (2013). Equalization of odor representations by a network of electrically coupled inhibitory interneurons. *Nat. Neurosci.* 16 1678–1686. 10.1038/nn.3528 24077563

